# Association between evergrande FC's club debt and Chinese super league's profitability from 2014 to 2019

**DOI:** 10.3389/fspor.2023.1095250

**Published:** 2023-02-27

**Authors:** Zeyong Liu, Yuqian Liu, Agudamu, Te Bu, Radenko Matic, Dusan Corilic, Catalina Casaru, Yang Zhang

**Affiliations:** ^1^ College of Physical Education, Hunan Normal University, Changsha, China; ^2^ School of Finance, Hunan University of Finance and Economics, Changsha, China; ^3^ Department of Social Welfare, College of Social Sciences, Sungkyunkwan University, Seoul, South Korea; ^4^ Faculty of Sport and Physical Education, University of Novi Sad, Novi Sad, Serbia; ^5^ Department of Physical Education and Athletic Training, University of West Alabama, Livingston, AL, United States; ^6^ Independent researcher, Orlando, FL, United States

**Keywords:** soccer, financing, sports industry, real estate, modern monetary theory

## Abstract

**Purpose:**

This study examined the role of Evergrande FC's club debt on the Chinese Super League (CSL)'s profitability from 2014 to 2019.

**Methods:**

We extracted the financial statements of Evergrande FC and evaluated its correlation with the profitability of CSL and Evergrande Group, which serves as a direct indicator of commercial growth.

**Results:**

The association between Evergrande FC's net loss and gross debt and CSL's profitability is positive, strong (all correlation coefficients > .89), and statistically significant (all *p* < .05). The association between Evergrande FC's net asset value and CSL's profitability is negative, strong (correlation coefficient = −.97), and statistically significant (*p* < .05).

**Conclusion:**

These data imply that there is a good likelihood of a causal relationship between the negative club returns generated by real estate investments and CSL's rapid commercial growth from 2014 to 2019. In essence, a dovish monetary-regulatory policy nexus during this period drove up the CSL premium. This business history from the sports industry is another illustration of how the modern coupling of economic leverage and regulatory policy could have asymmetric impacts on short-term market growth. Based on this debt-fueled business history, CSL should progress to a higher level of development in the future.

## Introduction

Compared to highly commercialized sports clubs in the United States and Europe, China's professional football (soccer) league is a latecomer to professional sports. In 1993, the ‘Club Constitution of the Chinese Football Association’ and the ‘Ten-Year Plan for Chinese Football Development’ were released, offering guidance for the professional development of Chinese football (1, 2). The official establishment of the Division A league in 1994 marked the beginning of reform efforts. The Division A league was renamed the Chinese Super League (CSL) in 2004. The Chinese Football Association and participating clubs jointly sponsored the foundation of CSL Limited Company in 2006, which is responsible for handling the league's match organization, commercial advertising, and copyright management. After nearly two decades of development, CSL has achieved major commercial progress. In the 2019 season, CSL has a viewership of approximately 700 million and sponsors from 13 different industries, generating a compound annual revenue of 614 million Yuan (3).

Throughout the world of professional football leagues, a large monetary commitment is required to ensure a club's success (4). Consequently, financial distress becomes a systemic issue not only in European football leagues (5) but also in China. Each CSL club needs at least 300 to 400 million Yuan in annual operating costs, and the championship club requires significantly more (6). To provide continual financial support, a strong capital base must exist, and in China, this capital base consists of real estate enterprises. Since the 1990s, China's economy has developed at an unprecedented rate, and real estate enterprises have advanced to the point that almost no industrial growth can match their dominance in CSL ownership. In the 2021 CSL season, 10 out of 16 CSL clubs were owned by real estate enterprises (Table 1) (7), showing that real estate capital remains the principal investor of CSL clubs.

**Table 1:** Shareholder structure of the Chinese super league clubs in 2021.

Club	Ownership
Qingdao FC	Shenzhen Hengye Investment Group Co., Ltd. owns 63.6%, Qingdao Huanghai Health Industry Group owns 27%, Qingdao Hainiu Jinhao Investment Co. Ltd. owns 6.3%; Qingdao Manatee Central Plaza Commercial Management Co., Ltd.* owns 3.1%
Changchun Yatai	Changchun Jialun Investment Management Co. owns 100%
Beijing Guoan	Sinobo Group* owns 64%; Citic Group owns 36%
Shandong Taishan	State Grid Shandong Electric Power Company owns 69.3%; Luneng Group owns 30.7%
Hebei FC	China Fortune Land Development Co., Ltd. Limited* owns 100%
Henan SSLM FC	Henan Hongdao Investment Co., Ltd. owns 98.6%; Henan Zhongyuan International Culture Communication Co., Ltd. owns 1.4%
Cangzhou Mighty Lions FC	Ever Bright Group* owns 50%; Cangzhou Construction Group* owns 50%
Wuhan FC	Wuhan Zall Culture & Tourism Group Co., Ltd. owns 100%
Shenzhen FC	Keyu Hotel Management Co., Ltd.* own 90%; Wan Hong-wei owns 10%
Dalian Pro	Yi Fang Group owns 95%; Dalian Yifang Real Estate Co., Ltd.* owns 5%
Tianjin Tigers	Tianjin Guohong Enterprise Management Co., Ltd.* owns 85.4%; Tianjin Economic and Technological Development Zone Investment Co., Ltd. owns 9.1%; Tianjin Beixin Asset Management Co., Ltd. owns 5.5%
Chongqing Liangjiang Athletic FC	Dangdai Group owns 90%; Lifan Motor Enterprises owns 10%
Shanghai Port	Shanghai International Port (Group) Co., Ltd. owns 100%
Shanghai Shenhua	Greenland Holdings Corporation Limited* owns 100%
Guangzhou City FC	R&F Properties* owns 100%
Guangzhou FC	China Evergrande Group* owns 60%; Alibaba Group owns 40%

* Indicate real estate capitals.

However, CSL's rapid but unstable rise has been noted, with its core characteristic being a massive capital monopoly. As Chinese real estate sales growth slows beginning in 2019 (8), the spillover effect is rapidly being exposed in CSL. CSL went through a quick expansion and contraction cycle, in terms of profitability, club operation, and league integrity. Guangzhou Evergrande Taobao FC (hereinafter referred to as Evergrande FC), majority owned by the China Evergrande Group (hereinafter referred to as Evergrande Group), was Asia and China's first publicly-traded football club in 2015 and became China's first delisted public sports company in 2020. CSL's sponsorship revenue plummeted to 308 million Yuan in 2020 (9), a fall of 50% from the previous season. Hebei FC, which was formerly controlled by China Fortune Land Development Co. Ltd. and once paid Argentina star Ezequiel Lavezzi a salary of 26.5 million euros (after tax) from 2016 to 2019 (10), has discontinued operations. Additionally, the 2021 season saw the withdrawal of the previous season's champion Jiangsu Suning FC from CSL due to insolvency. Such an unstable development model would harm the club and its players, as well as CSL's public image and long-term growth.

Despite this costly lesson for investors, CSL, and other interested parties, there have been few economic analyses of CSL's quick rise and fall. CSL clubs typically have a high debt-to-asset ratio. For example, according to Evergrande FC's financial statements, its debt-to-asset ratio increased from 73.87% in 2015 to 267.37% in 2019. During the same period, CSL's sponsorship revenue increased from 326 million Yuan (11) to 614 million Yuan. Because real estate enterprises in China often have to rely on debt to finance their operations (12), it is unsurprising that a league dominated by real estate enterprises is constructed on debt. Given this, the role of debt in CSL's financial performance is an interesting but previously unquantified topic.

Thus, the purpose of this study was to quantify the association between club debt and profitability of CSL and real estate enterprise. In this historical case analysis, we chose Evergrande FC and the 2014–2019 seasons as a representative sample and period for three reasons. First, Evergrande FC was a public company, which permitted an objective review of its financial statements. Second, between 2011 and 2019, Evergrande FC won the CSL eight times, the AFC Champions League twice, and the Chinese FA Cup twice. Evergrande FC is almost synonymous with CSL and was instrumental in the league's commercial development. Third, CSL and Evergrande Group achieved record earnings from 2014 to 2019, which rapidly declines after 2019.

## Methods

We extracted the financial statements of Evergrande FC and Evergrande Group between 2015 and 2019, covering the years 2014 through 2019. Data on CSL's profitability between 2014 and 2019 were published by CSL Limited Company and Deloitte (3, 11). The financial statements analyzed in this study are available on figshare (DOI: https://doi.org/10.6084/m9.figshare.21539232.v2).

The study quantified club debt using three metrics: net loss, gross debt, and net asset value. Profit or loss statements reflect a period's earnings performance, and sustained net loss generally results in deficits and rising debt. The net asset value equals total assets less gross debt, which provides additional insight into the financial health of a business.

CSL generates the majority of its income from sponsorship, broadcasting rights, and licensed merchandise products. We extracted revenue from sponsorship and broadcasting and analyzed the data in two ways. Sponsorship revenue acts as a dynamic indicator of revenue generation, reflecting annual fluctuations in the overall interest and investment in CSL. Second, we combined revenue from sponsorship and broadcasting. CSL signed a 10-year deal in 2015 and sold exclusive broadcasting rights valued at 11 billion Yuan. Evergrande Group's profitability was determined using the net income from its annual financial statements.

GraphPad 9.0 (GraphPad Software, Inc., United States) was used to analyze the data. Pearson correlation analysis was used to determine the association between Evergrande FC's debt and the profitability of CSL and Evergrande Group. A *p* < .05 was considered statistically significant. In addition, the magnitude of the correlation coefficient (*r*) was interpreted in accordance with Dancey and Reidy (13).

## Results

Between 2014 and 2019, Evergrande FC's net loss and gross debt climbed from 0.48 to 1.94 billion Yuan and 0.49 to 6.63 billion Yuan, respectively, while its net asset value decreased from 0.86 to −4.151 billion Yuan. During the same period, CSL's sponsorship revenue increased from 0.29 to 0.61 billion Yuan, and Evergrande Group's income increased from 18.02 to 33.5 billion Yuan.

**Figure 1:**
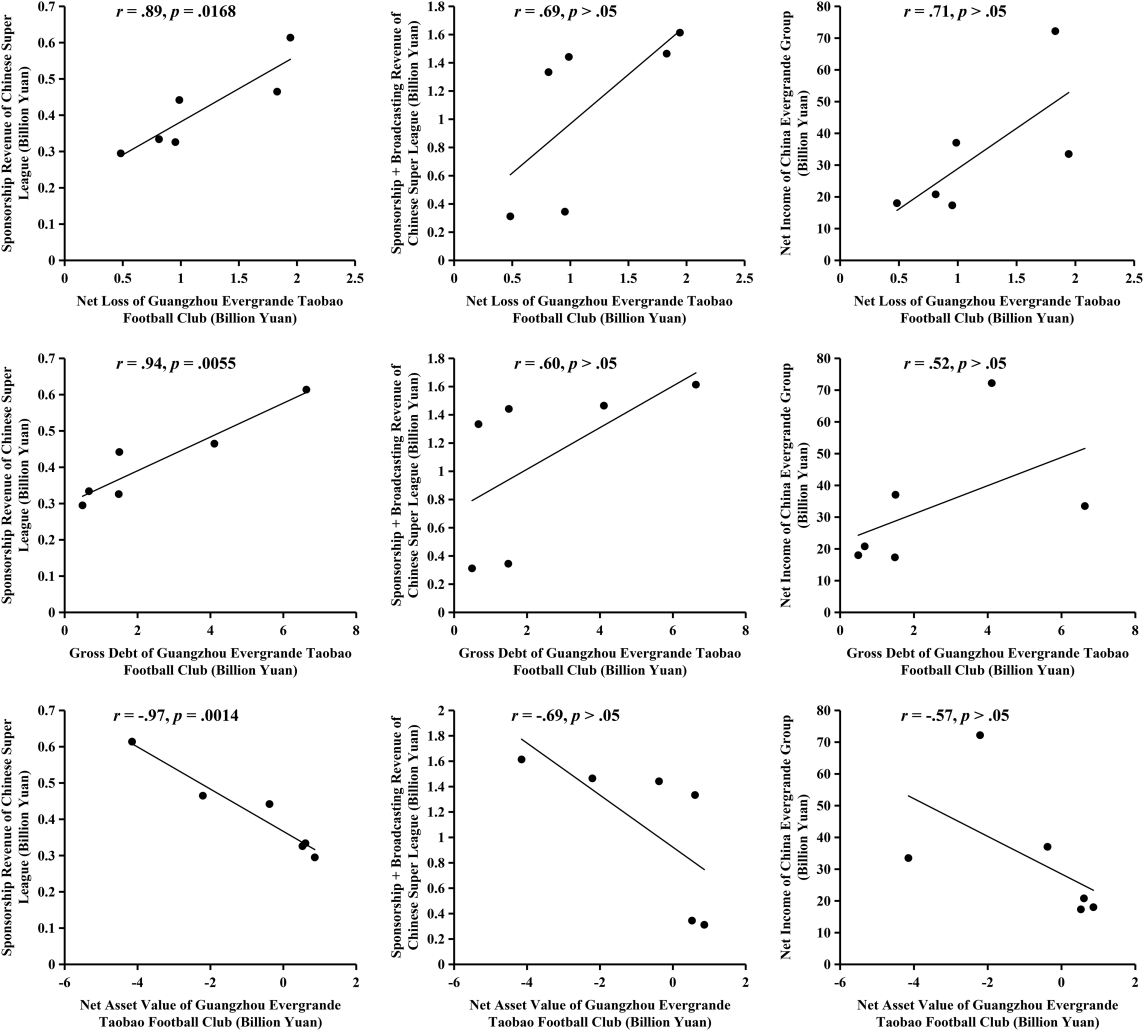
Association between evergrande FC's club debt and the profitability of the Chinese super league and evergrande group from 2014 to 2019.

Figure 1 shows the correlation between Evergrande FC's debt and the profitability of CSL and Evergrande Group. There is a positive, strong (*r* = .89), and significant (*p* < .05) correlation between Evergrande FC's net loss and CSL's sponsorship revenue. There is a positive, moderate, but non-significant correlation between Evergrande FC's net loss and CSL's sponsorship and broadcasting revenue. There is a positive, strong, but non-significant correlation between Evergrande FC's net loss and Evergrande Group's net income.

There is a positive, strong (*r* = .94), and significant (*p* < .05) correlation between Evergrande FC's gross debt and CSL's sponsorship revenue. There is a positive, moderate, but non-significant correlation between Evergrande FC's gross debt and CSL's sponsorship and broadcasting revenue, and Evergrande Group's net income.

There is a negative, strong (*r* = -.97), and significant (*p* < .05) correlation between Evergrande FC's net asset value and CSL's sponsorship revenue. There is a negative, moderate, but non-significant correlation between Evergrande FC's net asset value and CSL's sponsorship and broadcasting revenue, and Evergrande Group's net income.

## Discussion

In this study, we show that, as Evergrande FC's debt accumulated, CSL's revenue-generating ability continued to increase. In Europe, it has been found that investors in football clubs neither expect nor demand a financial return (14). In the East, massive capital investments from Evergrande FC and other CSL clubs backed by real estate firms boosted the CSL's popularity (9, 15). We argue that the case for a causal effect running from high club debt to strong CSL growth in the examined period is highly probable. This pattern has been observed in non-sports empirical studies finding a favorable association between debt increase and economic growth (16, 17). This is the first time that the favorable effect of debt on the development of commercial sports has been observed in China.

It has been revealed that real estate enterprises that invest in CSL clubs generate some of the highest corporate earnings and sales in the same industry (18). The real estate industry requires a large investment in projects, and a strong policy influence over bank financing, land auctions, and project approval, making it critical to obtain low-cost credit and attractive land plots with policy support. The smooth establishment of development projects is critical for real estate enterprises' capital flow and profitability. For real estate enterprises, it makes sense to invest in CSL clubs in a particular region. Previously Division A clubs, and now CSL clubs, have been developed in the region for many years and have a high degree of regional integration. Meanwhile, local governments leverage CSL clubs to promote the city's diversification and therefore provide policy support in terms of land acquisition, taxation, and bank financing for real estate enterprises that commit to investing in CSL clubs (19). This has become an important reason for real estate enterprises to enter the football arena (20). Thus, a large amount of real estate capital has been invested in CSL, which can foster a positive relationship between institutional support and business interests.

In our opinion, an important factor fueling CSL's profitability from 2014 to 2019 is China's unique monetary-regulatory policy nexus during this period. Since its inception, CSL has followed the British model of vertical hierarchical governance, and each club's operation invariably relies on the input of investors (21). Private capital investment in China is essentially a targeted reallocation of central bank credit (22), and real estate enterprises cannot invest large sums without expansionary monetary policy, let al.one a loss-making venture such as Evergrande FC. Between December 2014 and December 2015, China's reserve requirement ratio was lowered by 300 basis points, and the monetary easing phase continued until June 2018 (23), followed by a flourishing real estate industry (24). Thus, a critical transmission channel is the inflow of cheap bank loans to real estate enterprises, which enables them to fuel investment in CSL clubs and CSL's booming business.

In addition, the Chinese government has sought to develop a professional football management system and operational mechanism, and the 2015 ‘Overall Plan for Chinese Football Reform and Development’ charts the course for CSL's market principles (2). Because football reform has become a national commitment for contemporary Chinese society, promotion of leisure activities, and all-round development of Chinese youth (25), it is critical for both public and private sectors to support youth and professional football development at all levels. Local governments have policy responsibilities to prioritize football in society, and regulatory preferences have been granted to the real estate industry, which controls enormous capital. As a result, the real estate industry has evolved into an ‘invisible’ financier of social transformation.

Management of professional football clubs typically requires clubs to operate on a balanced budget to ensure long-term financial viability, as promoted by the UEFA Financial Fair Play Regulations. However, the European experience has demonstrated a tenuous link between increasing financial stability and club profitability (26, 27), resulting in a fall in the competitive balances of leagues like Spain, Germany, and France (28, 29). From a broader view, the policy tilt toward reducing fiscal deficits and debt levels had produced a negative spiral of weak growth and rising inequality (28). We argue that debt financing supported CSL's profitability and growth from 2014 to 2019 and that its beneficial effect on modern social transition extends far beyond the narrow lens of balance sheet deficit. Modern monetary theory refutes the assumption that deficits and debt should be avoided at all costs (30). Instead, the efficiency of capital expenditures and return should be judged in terms of the actual resources available for productive use in human society. Between 2014 and 2019, monetary transfer from the real estate industry to CSL left a plethora of legacies for Chinese society, including women and youth football involvement (9, 31), upgraded consumer sports consumption (32), and modern urbanization (20). The Chinese Communist Party's success is premised on placing the interests of the people-centered. CSL is therefore more than just a sport or a business in China. A healthy Chinese population in the 21st century is a critical component of the ‘Chinese Dream’ (33), and football plays a vital role in accomplishing this goal (34).

Our arguments should not be interpreted as implying that excessive debt is not a serious problem. The domestic and international macro environment evolves rapidly, and debt-financed growth must respond consistent with updated monetary and regulatory policies. Despite the current analysis covering the period from 2014 to 2019, the policy shifts beginning in late 2018 are critical for summarizing the experiences, and we briefly explain two hawkish policy shifts that plunge CSL into contraction. In response to the trade war, in July 2018, China refined its coordination mechanisms to stabilize six key areas, including employment, financial, foreign trade, investment, and market expectations (24). Consequently, monetary policy tightened fast to reduce exposure in the heavily leveraged real estate industry. Since December 2018, shadow rates have tightened substantially from cycle lows, and real estate entrusts, which issued shadow banking instruments and garnered considerable funds for the real estate industry's operations, including football investment, have plummeted (35). Meanwhile, in 2019, the Chinese Football Association implemented a series of regulatory frameworks, including strict wage limits (15), club names that must be not commercial (36), and club equity restructuring (37). These restrictive regulatory policies further amplified the negative effect of tightening monetary policy, thus crowding real estate enterprises out of CSL.

In conclusion, we argue that debt had an asymmetric effect on CSL's profitability from 2014 to 2019 and that its beneficial impact on Chinese society may be far greater than can be quantified through simple metrics such as corporate deficits. CSL's recent contraction is a direct outcome of contractionary monetary and regulatory policies, and so there was scope for a rapid recovery if the capital investment can be better regulated and planned. Meanwhile, the reappearance of the COVID-19 pandemic in 2022 could be a delicate juncture for policymakers to pursue an expansionary fiscal strategy to stimulate the high-quality, structural transformation of Chinese sports (38) and economy (39).

## Data Availability

The datasets presented in this study can be found in online repositories. The names of the repository/repositories and accession number(s) can be found in the article/Supplementary Material.
